# Presence and extent of cardiac computed tomography angiography defined coronary artery disease in patients presenting with syncope

**DOI:** 10.1007/s12471-017-0970-7

**Published:** 2017-03-20

**Authors:** S. Altintas, T. Dinh, N. G. H. M. Marcks, M. Kok, A. J. J. Aerts, B. Weijs, Y. Blaauw, J. E. Wildberger, M. Das, B. L. J. H. Kietselaer, H. J. G. M. Crijns

**Affiliations:** 1 Department of Cardiology, Maastricht University Medical Center+ (MUMC+), Maastricht, The Netherlands; 2 Cardiovascular Research Institute Maastricht (CARIM), School for Cardiovascular Diseases, MUMC+, Maastricht, The Netherlands; 3 Department of Radiology, MUMC+, Maastricht, The Netherlands; 4 Department of Cardiology, Zuyderland Medical Center, Heerlen, The Netherlands; 5 Department of Cardiology, University Medical Center Groningen, Groningen, The Netherlands

**Keywords:** Coronary artery disease, Multidetector computed tomography, Cardiogenic syncope

## Abstract

**Background:**

In syncope patients, presence of coronary artery disease (CAD) is associated with poor prognosis. However, data concerning CAD prevalence in syncope patients without known cardiovascular disease are lacking. Therefore, the aim of this study was to investigate presence and extent of CAD in syncope patients.

**Methods:**

We included 142 consecutive patients presenting with syncope at the outpatient cardiology clinic who underwent coronary computed tomography (CT) angiography. Syncope type was ascertained by two reviewers, blinded for coronary CT angiography results. Of the patients, 49 had cardiac syncope (arrhythmia or structural cardiopulmonary disease) and 93 had non-cardiac syncope (reflex [neurally-mediated], orthostatic or of unknown cause). Cardiac syncope patients were compared with matched stable chest pain patients regarding age, gender, smoking status, diabetes mellitus type 2 and systolic blood pressure.

**Results:**

Distribution of CAD presence and extent in cardiac and non-cardiac syncope patients was as follows: 72% versus 48% any CAD; 31% versus 26% mild, 8% versus 14% moderate and 33% versus 7% severe CAD.

Compared with non-cardiac syncope, patients with cardiac syncope had a significantly higher CAD presence and extent (*p* = 0.001). Coronary calcium score, segment involvement and stenosis score were also higher in cardiac syncope patients (*p*-values ≤0.004). Compared to the chest pain control group, patients with cardiac syncope showed a higher, however, non-significant, prevalence of any CAD (72% versus 63%) and severe CAD (33% versus 19%).

**Conclusion:**

Patients with cardiac syncope show a high presence and extent of CAD in contrast to non-cardiac syncope patients. These results suggest that CAD may play an important role in the occurrence of cardiac syncope.

**Electronic supplementary material:**

The online version of this article (doi: 10.1007/s12471-017-0970-7) contains supplementary material, which is available to authorized users.

## Introduction

Syncope is defined as a transient loss of consciousness due to transient global cerebral hypoperfusion and is characterised by a rapid onset, short duration and complete spontaneous recovery [1, 2]. It concerns a common medical problem, with an incidence rate of 6.2 per 1000 person-years for a first report [3]. Syncope is a symptom with a wide spectrum of potential aetiologies for which accurate diagnosis, using additional testing, is of high importance [4–6]. Along with the search for the underlying diagnosis, defining prognosis is crucial, whereby the risk of death, recurrence, as well as life-threatening events should be considered [2, 4].

Within the current European Society of Cardiology Guidelines for the diagnosis and management of syncope, only limited guidance is offered for diagnostic strategies to detect CAD [2]. Ischaemia evaluation is recommended within the current guidelines of the American College of Cardiology/American Heart Association (ACC/AHH) for syncope patients with known, or who are at risk for, coronary artery disease (CAD) [6]. However, there is no guidance regarding anatomical imaging techniques to detect CAD in syncope patients despite the evidence that arrhythmic causes for syncope, such as atrial fibrillation or ventricular tachycardia, have a higher risk of major adverse cardiovascular and cerebral events in the presence of CAD [7, 8]. Additionally, all patients with syncope and ischaemic heart disease have an increased risk of death [1, 3, 9].

Currently, coronary computed tomography (CT) angiography (CCTA) is a widely implemented non-invasive imaging modality to diagnose CAD [10–12]. Conventional CCTA reading includes assessment of the coronary calcium score (CCS), luminal stenosis severity and extent of CAD with high sensitivity and specificity [10, 12]. It may be considered in stable chest pain patients with an intermediate pre-test probability of ischaemic heart disease [13]. Despite the wide use of CCTA in patients with stable chest pain, there are no recommendations for CAD detection with CCTA in patients presenting with syncope. Nevertheless, diagnosing CAD within syncope patients in an early stage could have important prognostic and therapeutic clinical implications. Therefore, the aim of the present study was to investigate the presence and extent of CAD, as defined by CCTA, in patients presenting with syncope at the outpatient cardiology clinic.

## Methods

### Study population

This was an observational single-centre study analysing 142 retrospectively collected consecutive patients presenting with syncope at the outpatient cardiology department between May 2007 and April 2015 who were referred for CCTA within their diagnostic workup. Patients were selected if they met the definition of syncope, which was defined as transient loss of consciousness due to global cerebral hypoperfusion with rapid onset, short duration and spontaneous complete recovery [2].

General exclusion criteria for CCTA examination were: haemodynamic instability, pregnancy, renal insufficiency (defined as glomerular filtration rate <45 ml/min/1.73 m) and known severe allergic reactions regarding iodine.

This study was approved by the Institutional Review Board (METC 15-4-091) and complies to the ethical guidelines of the 1975 Declaration of Helsinki. Written informed consent was waived, because the data were anonymously recorded and analysed in accordance with guidelines of our Review Board.

### Data collection and definitions

Data regarding syncope type, age, gender, cardiovascular risk factors, medication use, additional diagnostic testing and CCTA results were collected from the patients’ charts and electronic medical records.

The type of syncope was ascertained by chart review by two reviewers (N.M., T.D.), blinded for CCTA results. The definitive syncope type was ascertained by consensus between the two reviewers. The following pathophysiological classification and sub-classification was used to adequately define the syncope types in each individual patient [2]:

1. *Cardiac syncope (cardiovascular)*: arrhythmia or structural cardiopulmonary disease as primary cause,
2. *Reflex (neurally-mediated) syncope*: vasovagal, situational or carotid sinus syncope,
3. *Syncope due to orthostatic hypotension*: primary or secondary autonomic failure, drug-induced orthostatic hypotension or volume depletion,
4. *Syncope of unknown cause: *defined as an unknown cause despite additional diagnostic testing.

Subsequently, patients with *reflex syncope, orthostatic syncope* and *syncope of unknown cause* were classified as having ‘non-cardiac syncope’, which led to two main syncope categories: *cardiac *and *non-cardiac syncope*.

Diabetes mellitus was defined as fasting glucose levels of ≥7 mmol/l or treatment with either diet intervention, oral glucose lowering agent or insulin [14]; smoking was defined as current smoking. A positive family history was defined as having a first-degree relative with a history of myocardial infarction or sudden cardiac death before the age of 60.

The PROCAM risk score was determined according to the following parameters: age, LDL cholesterol, smoking, HDL cholesterol, systolic blood pressure, family history of premature myocardial infarction, diabetes mellitus, and triglycerides [15]. This risk score predicts the absolute 10-year risk for the occurrence of an acute coronary event (fatal or non-fatal myocardial infarction or acute coronary death). A score <10% is estimated as low risk, 10–20% as intermediate risk, and >20% as high risk.

### Control population

To further study the impact of cardiac syncope symptoms on detection of CAD, we compared the cardiac syncope patients with stable chest pain patients. The cardiac syncope patients were compared (1:2 ratio) with stable chest pain patients, referred for CCTA from the outpatient cardiology clinic to compare the prevalence and extent of CAD in cardiac syncope patients with stable chest pain patients. Matching was based upon age, gender, smoking status, diabetes mellitus type 2 and systolic blood pressure (±10 mm Hg).

### CCTA acquisition

Scans were performed using a 64-slice multidetector computed tomography scanner (Brilliance 64; Philips Healthcare, Best, The Netherlands) or a 2^nd^ generation dual-source CT scanner (Somatom Definition Flash, Siemens Healthcare, Forchheim, Germany). Data acquisition parameters for the Brilliance 64 were a 64 × 0.625 mm slice collimation, a gantry rotation time of 420 ms and a tube voltage of 80 or 120 kV depending on patients’ height and weight. Data acquisition parameters for the Somatom Definition Flash were a 2 × 128 × 0.600 mm slice collimation, a gantry rotation time of 280 ms and a tube voltage of 100 or 120 kV depending on patients’ height and weight. A non-contrast enhanced scan was performed using 120 kV and 3 mm slice thickness to determine the CCS [16]. CCTA was performed using 75–120 ml of contrast agent (Xenetix 350; Guerbet, France or Ultravist 300; Bayer Healthcare, Berlin, Germany) injected in the antecubital vein at a rate of 5.2–7.4 ml/s followed by 40 ml intravenous saline at the same flow rates.

Scan protocols differed between both CT scanners. For the 64-slice scanner, a prospectively gated ‘Step and shoot’ protocol was used in patients with stable heart rate <65 bpm. In patients with a heart rate >65 bpm, a retrospectively gated ‘Helical’ protocol was used with dose modulation. For the 2^nd^ generation dual-source CT scanner, a prospectively gated high-pitch spiral ‘Flash’ protocol was used in patients with a stable heart rate <60 bpm. In patients with a stable heart rate between 60–90 bpm, a prospectively gated axial ‘Adaptive sequence’ protocol was used. In patients with a heart rate >90 bpm or with an irregular heart rhythm, a retrospectively gated ‘Helical’ protocol was used. Dose modulation was switched on in all three protocols using tube current modulation (CARE Dose4D, Siemens Healthcare, Forchheim, Germany).

Patients received 50 mg Metoprolol tartrate orally (AstraZeneca, Zoetermeer, the Netherlands), two hours before CCTA, unless contra-indicated. If indicated, an additional dose of 5–20 mg Metoprolol was administered intravenously to lower the heart rate. All patients received nitroglycerine (Pohl-Boskamp, Hohenlockstedt, Germany) sublingually in a dose of 0.8 mg just prior to CCTA.

### CCTA assessment

The Agatston method was used to define the CCS [16]. CCTA’s were independently analysed by a cardiologist and a radiologist, both experienced in the assessment of CCTA. In case of disagreement, consensus was reached by discussion. The assessment was performed using the source images on the provided software (Cardiac Comprehensive Analysis, Philips Healthcare or Syngo CT 2010A, Siemens Healthcare). The coronary artery tree was analysed for the presence and extent of CAD, according to the 16-segment classification of the American Heart Association [17]. Plaques were defined as visible structures within or adjacent to the coronary artery lumen, which could be clearly distinguished from the vessel lumen and the surrounding pericardial tissue. The degree of stenosis was visually defined and classified as no CAD (no luminal stenosis), mild (<50% luminal stenosis), moderate (50–70% luminal stenosis), severe (>70% luminal stenosis). A segment involvement score was defined by counting all coronary segments with plaques (irrespective of degree of stenosis), which resulted in a score ranging from 0–16 [18]. A segment stenosis score was the sum of the lesion severity in all 16 coronary segments, resulting in a score ranging from 0–48 [18].

### Statistical analysis

Data were analysed using SPSS version 23.0 (SPSS Inc., Chicago, IL, USA). Continuous variables were checked whether they were normally distributed using box plots, histograms or by computing skewness and kurtosis. Continuous data were reported as means and standard deviations (SDs) if normally distributed. If data were not normally distributed, continuous data were reported as medians and boundaries of interquartile ranges (IQR). Proportions (%) were used for categorical values.

Differences across groups were assessed using the independent *t*-test for normally distributed data after performing Levene’s test for equality of variance. The Mann-Whitney test was used for data that were not normally distributed. Categorical variables were tested with Fisher’s exact test.

All *p-*values were 2‑sided, and a *p*-value below 0.05 was considered statistically significant.

## Results

### Study population

Between May 2007 and April 2015, 142 consecutive patients presented with syncope at the outpatient cardiology clinic who were referred for CCTA. The distribution of syncope classifications was as follows: 49 (35%) cardiac syncope, 63 (44%) reflex (neurally-mediated) syncope, 10 (7%) orthostatic syncope and 20 (14%) syncope of unknown cause. Subsequently, patients with reflex syncope, orthostatic syncope and syncope of unknown cause were classified as having ‘non-cardiac syncope’ leading to two main categories, with 49 (35%) cardiac syncope patients and 93 (65%) non-cardiac syncope patients.

The baseline characteristics of the study population are described in Table 1. When compared to non-cardiac syncope patients, only age showed a statistically significant difference between the cardiac and non-cardiac syncope groups (mean (SD): 60 (13) versus 54 (12); *p* = 0.002). *The Online Supplemental Material* provides further detailed clinical information regarding the syncope patients.

**Table 1:** Baseline characteristics of the study population

	Cardiac syncope vs non-cardiac syncope *n =* 142			Cardiac syncope vs chest pain control group *n =* 147		
Patient characteristics	Cardiac syncope *n = 49*	Non-cardiac syncope *n = 93*	*P*-value	Cardiac syncope *n = 49*	Chest pain control group *n = 98*	*P*-value
Age, *years*	60 ± 13	54 ± 12	**0.002**	60 ± 13	60 ± 12	0.810
Male gender	32 (65)	57 (61)	0.716	32 (65)	64 (65)	>0.999
BMI, *kg/m* ^2^	26 ± 3	26 ± 4	0.365	26 ± 3	27 ± 4	0.116
Systolic Blood Pressure, *mm Hg*	143 ± 20	140 ± 21	0.349	143 ± 20	144 ± 16	0.990
Diastolic Blood Pressure, *mm Hg*	81 ± 13	83 ± 13	0.396	81 ± 13	78 ± 9	0.140
Active smoking	19 (39)	27 (29)	0.256	19 (39)	38 (39)	>0.999
Diabetes mellitus II	3 (6)	4 (4)	0.693	3 (6)	5 (5)	>0.999
Family history of CAD	16 (33)	43 (46)	0.152	16 (33)	27 (28)	0.566
PROCAM risk score	9 (4–21)^a^	7 (2–16)^b^	0.131	9 (4–21)^a^	8 (3–16)	0.280

Continous variables are described as mean (±SD) or as median (interquartile range); categorical variables as number (%)

*BMI* body mass index

^a^ *n* = 19 lost to PROCAM risk score; ^b^ *n* = 34 lost to PROCAM risk score

### Presence and extent of CAD in syncope

The distribution of CAD presence and extent in cardiac and non-cardiac syncope patients was as follows: 72% versus 48% any CAD; 31% versus 26% mild CAD; 8% versus 14% moderate CAD and 33% versus 7% severe CAD (Table 2)*.*

**Table 2:** Distribution of conventional CT parameters across cardiac syncope patients versus non-cardiac syncope patients and matched chest pain control group

	Cardiac syncope vs non-cardiac syncope *n = 142*			Cardiac syncope vs chest pain control group *n = 147*		
CT parameters	Cardiac syncope *n = 49*	Non-cardiac syncope *n = 93*	*P*-value	Cardiac syncope *n = 49*	Chest pain control group *n = 98*	*P*-value
Presence and extent of CAD	–	–	**0.001**	–	–	0.133
– no CAD	14 (28)	49 (53)	**–**	14 (28)	36 (37)	***–***
– mild CAD, *<50% luminal stenosis*	15 (31)	24 (26)	**–**	15 (31)	25 (26)	***–***
– moderate CAD, *50–70% luminal stenosis*	4 (8)	13 (14)	**–**	4 (8)	18 (18)	***–***
– severe CAD, *>70% luminal stenosis*	16 (33)	7 (7)	**–**	16 (33)	19 (19)	***–***
Coronary calcium score, *AU*	80 (0–387)	0 (0–79)	**0.002**	80 (0–387)	36 (0–205)	0.272
Segment involvement score, *0–16*	2 (0–5)	0 (0–2)	**0.004**	2 (0–5)	2 (0–4)	0.364
Segment stenosis score, *0–48*	3 (0–7)	0 (0–3)	**0.003**	3 (0–7)	2 (0–7)	0.477

*CT* computed tomography, *CAD* coronary artery disease, *AU* Agatston Unit

**Fig. 1:**
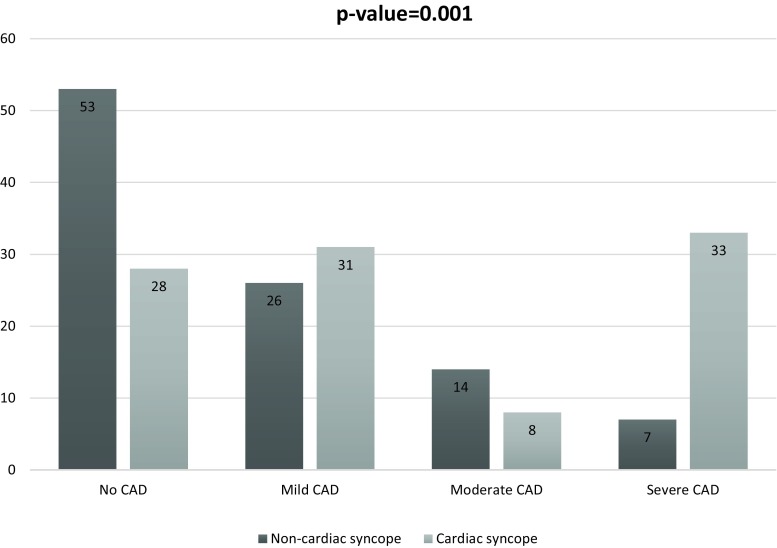
CAD presence and extent (percentages) within cardiac syncope patients versus non-cardiac syncope patients

Fig. 1 visualises CAD prevalence and distribution of CAD in patients with cardiac and non-cardiac syncope. CAD presence and extent was significantly higher in patients with cardiac syncope in comparison with non-cardiac syncope patients (Table 1;* p* = 0.001). Additionally, all conventional CT parameters including CCS, segment involvement and segment stenosis score were significantly higher in patients with cardiac syncope compared to non-cardiac syncope (80 [0–387] versus 0 [0–79]; 2 [0–5] versus 0 [0–2]; 3 [0–7] versus 0 [0–3]) (all* p*-values ≤0.004; Table 2).

### Presence and extent of CAD in patients with cardiac syncope versus matched chest pain controls

**Fig. 2:**
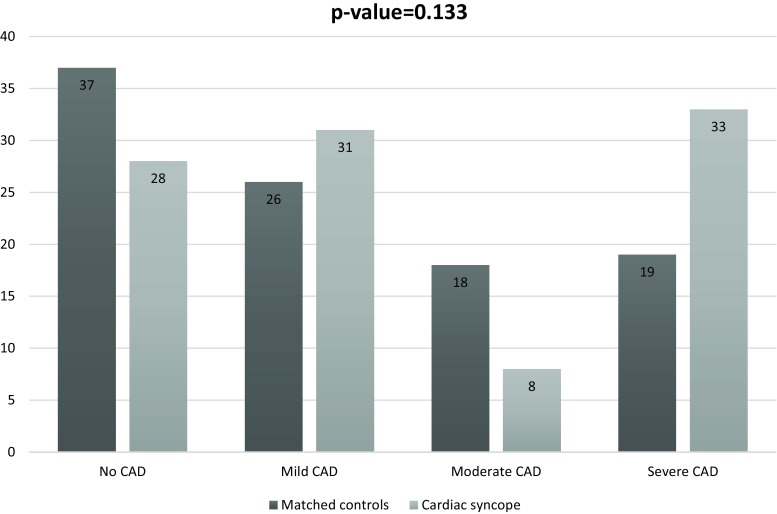
CAD presence and extent (percentages) within cardiac syncope versus matched chest pain control group

Fig. 2 displays the presence and extent of CAD in both cardiac syncope patients and matched chest pain controls. Patients with cardiac syncope showed a higher prevalence of any CAD (72% versus 63%, respectively) and percentage of severe luminal stenosis (33% versus 19%). Interestingly, no statistically significant difference was observed between cardiac syncope patients and chest pain controls regarding overall prevalence of CAD presence and its extent (*p* = 0.133).

Table 2 shows that all other conventional CT parameters including CCS, segment involvement score and segment stenosis score were comparable between cardiac syncope patients and chest pain controls (all *p*-values ≥0.272).

## Discussion

To our best knowledge, this is the first study dedicated to investigating the prevalence and extent of CAD in patients presenting with syncope. The main finding of this study was that cardiac syncope patients showed a high presence and extent of CAD in comparison with non-cardiac syncope patients. In addition, all other coronary CT parameters like CCS, segment involvement score and stenosis score were also significantly higher in cardiac syncope patients compared to non-cardiac syncope patients. When compared to stable chest pain controls, patients with cardiac syncope showed a higher, however, non-significant, prevalence of any CAD (72% versus 63%, respectively) and percentage of severe luminal stenosis (33% versus 19%). Taken together, these results suggest that the non-invasive evaluation of CAD, using CCTA, could be considered within the diagnostic workup of patients presenting with cardiac syncope at the outpatient cardiology clinic. Additionally, CCTA may show alternative causes for cardiovascular syncope such as congenital anomalies of coronary arteries, hypertrophic cardiomyopathy, pulmonary embolism, obstructive valvular heart disease, intracardiac masses and pericardial diseases.

### Association and relevance of CAD in cardiac syncope

Meticulous history taking is essential in arriving at a diagnosis in patients with syncope. Once cardiac syncope is suspected, a wide range of different diagnostic tests can be considered in patients presenting with syncope [2, 6]. However, there is no guidance regarding anatomical imaging techniques to detect CAD due to lacking evidence describing the extent and nature of CAD in patients with cardiac syncope. The relationship between cardiac ischaemia and syncope is multiple, including induction of non-sustained ventricular arrhythmias and sinoatrial or atrioventricular block, or by triggering e. g. the Bezold-Jarisch-reflex causing severe bradycardia and hypotension. Apart from a direct relationship, indirect mechanisms may be important, including old myocardial infarction with ventricular remodelling as a basis for re-entrant or adrenergic ventricular tachycardia; likewise, atrial remodelling leading to late onset atrioventricular nodal tachycardia or atrial tachycardias may at times occur with well-known haemodynamic compromise eliciting syncope at the beginning of the attack [19]. In all of the above-mentioned aetiologies, CAD may be causal or contributory for syncope whereby one could conjecture that interventional and vascular prophylactic management may help to reduce further syncope in these patients. In all other cases, CAD presence should be considered as coincidental wherefore vascular prophylactic vascular management may not be indicated in the management of syncope.

### Current guidance for the detection of CAD in cardiac syncope

Within the European Society of Cardiology guidelines for the diagnosis and management of syncope, exercise stress testing is only recommended in patients with suspected exercise-induced syncope, which concerns a rare condition [2]. Concurrently, ischaemia evaluation is recommended by the American College of Cardiology/American Heart Association (ACC/AHA) for patients with syncope and an intermediate-to-high risk for coronary heart disease or known CAD, but such strategy may underdiagnose the presence of non-obstructive CAD, which still is associated with high major adverse cardiac event rates [6, 20, 21]. On the other hand, a previous report revealed a low-diagnostic yield for stress myocardial perfusion imaging across all risk categories in syncope patients without known CAD [22].

## Prior studies

Previously, Soteriades et al. investigated the incidence and prognosis of syncope among participants of the Framingham Heart Study and found that patients with a cardiac syncope were more likely to have a history of CAD and were at an increased risk for death from any cause and cardiovascular events [3]. A more recent study of patients presenting with syncope at the emergency department with trauma, has shown that patients with a history of CAD are four times more likely to have cardiac syncope in contrast to non-cardiac syncope [23].

These previous reports support the high prevalence and extent of CAD in patients presenting with cardiac syncope compared to patients with non-cardiac syncope within the present study. Furthermore, in the presence of obstructive CAD in patients with syncope, treatment by either percutaneous coronary intervention (PCI) or medical management did not improve readmission rates due to syncope. However, PCI did improve long-term mortality in patients with syncope, suggesting the need for imaging of the coronary arteries [24].

Moreover, in syncope patients with left ventricular dysfunction, inducible ventricular tachycardia was frequent in the presence of CAD and associated with a bad prognosis [7]. Therefore, by diagnosing stable CAD and providing additional treatment with vascular protective medication, the prognosis of cardiac syncope patients could be positively influenced. HMG-CoA reductase inhibitors (statins) have become a cornerstone in the treatment of patients with stable CAD due to their lipid-lowering characteristics and additional atherosclerotic plaque stabilization, systemic inflammation and thrombogenicity reducing effects [25]. In line with these findings, recent review articles summarise that statins even reduce the incidence of ventricular tachycardia/fibrillation and sudden cardiac death in patients with CAD due to their anti-ischaemic, and possibly also by their antiarrhythmic or anti-inflammatory, effects [26, 27].

## Study limitations

This study has several limitations that should be mentioned. Firstly, it concerns a study with a relatively small sample size. Secondly, there was some degree of referral bias considering that our institution is a tertiary centre for patients with syncope. This is confirmed by the fact that within our syncope study population, a higher relative prevalence of cardiac syncope was observed in comparison to previous reports [2, 3]. Thirdly, the syncope patients were included if they were referred for CCTA, inducing some degree of selection bias. The combination of referral as well as selection bias could have contributed to the high prevalence and extent of CAD within the cardiac syncope patients. Also important is the fact that no direct causal relationships could be identified regarding the presence and extent of CAD and syncope due to the present study design, as this would require a prospective interventional study.

## Conclusions

Patients with cardiac syncope show a high presence and extent of CCTA defined CAD in contrast to patients with non-cardiac syncope. These results suggest that CAD may play an important role in the occurrence of cardiac syncope and should be considered in the diagnostic workup and treatment of syncope patients.

## Caption Electronic Supplementary Material

Supplementary Table 1 Detailed characteristics of syncope patients
