# Supplementary material for: Presence and extent of cardiac computed tomography angiography defined coronary artery disease in patients presenting with syncope
Source: Neth Heart J. 2017 Mar 20;25(6):376–87. doi: 10.1007/s12471-017-0970-7 (PMC5435619; doi:10.1007/s12471-017-0970-7)
Supplement: Supplementary file 1 — Supplementary Table 1 Detailed characteristics of syncope patients [file 12471_2017_970_MOESM1_ESM.docx]

| **Patient characteristics** | **Total**  ***n=142*** | **Cardiac syncope**  ***n=49*** | **Non-cardiac syncope**  ***n=93*** | **P-value** |
| --- | --- | --- | --- | --- |
| ***Medication use*** |  | | | |
| Antihypertensive agent | 53 (37) | 24 (49) | 29 (31) | **0.045** |
| Statin | 29 (20) | 12 (25) | 17 (18) | 0.390 |
| ACEi/ARB | 27 (19) | 14 (29) | 13 (14) | **0.044** |
| Beta blocker | 29 (20) | 11 (22) | 18 (19) | 0.667 |
| Calcium channel blocker | 11 (8) | 5 (10) | 6 (7) | 0.513 |
| Diuretic | 14 (10) | 5 (10) | 9 (10) | >0.999 |
| Antiplatelet | 21 (15) | 13 (27) | 8 (9) | **0.006** |
| Antiarrhytmic agent | 4 (3) | 3 (6) | 1 (1) | 0.119 |
| ***Electrocardiography*** |  | | | |
| Heartrate, *bpm* | 72 (62 – 82) | 73 (60 – 85) | 71 (63 – 79) | 0.610 |
| P-wave duration, *msec* | 80 (80-80) | 80 (80 - 80) | 80 (70 – 80) | 0.216 |
| PQ-interval duration, *msec* | 156 ± 23 | 158 ±24 | 154 ± 22 | 0.326 |
| QRS-duration, *msec* | 92 (84 – 102) | 90 (82 – 100) | 92 (84 – 104) | 0.639 |
| QTc-duration, *msec* | 423 (405 – 445) | 426 (416 – 452) | 417 (400 – 439) | **0.016** |
| ***Echocardiography*** |  | | | |
| LVEF, *%* | 60 ± 6 | 60 ± 8 | 59 ± 6 | 0.821 |
| LA volume, *ml* | 63 ± 17 | 64 ± 20 | 62 ± 16 | 0.584 |
| LV mass, *mg* | 155 ± 37 | 155 ± 42 | 155 ± 33 | 0.969 |
| IVST, *mm* | 9 (8 – 9) | 9 (8 – 10) | 9 (8 – 9) | 0.870 |
| PWT, *mm* | 8 (8 – 9) | 8 (8 – 9) | 9 (8 - 9) | 0.103 |
| *Aortic valve disease* |  | | | 0.061 |
| Sclerosis | 16 (13) | 7 (16) | 9 (11) |  |
| Stenosis | 3 (2) | 3 (7) | 0 |  |
| Insufficiency | 10 (8) | 5 (11) | 5 (6) |  |
| Bicuspidy | 3 (2) | 2 (5) | 1 (1) |  |
| *Mitral valve disease* |  | | | 0.144 |
| Stenosis | 0 | 0 | 0 |  |
| Insufficiency | 6 (5) | 4 (9) | 2 (2) |  |
| *Tricuspid valve insufficiency* | 3 (2) | 1 (2) | 2 (2) | 0.347 |
| ***Additional testing*** |  | | | |
| Cardiac stress test | 127 (89) | 45 (92) | 82 (88) | 0.578 |
| Holter | 121 (85) | 42 (86) | 79 (85) | >0.999 |
| Electrophysiology study | 16 (11) | 10 (20) | 6 (7) | **0.023** |
| Implantable Loop Recorder | 31 (22) | 19 (39) | 12 (13) | **0.001** |
| Cardiac MRI | 9 (6) | 4 (8) | 5 (5) | 0.496 |
| Invasive Coronary Angiography | 30 (21) | 17 (35) | 13 (14) | **0.005** |
| Tilt table test | 23 (16) | 4 (8) | 19 (20) | 0.092 |
| Electroencephalography | 11 (8) | 1 (2) | 10 (11) | 0.097 |
| MRI Cerebrum | 14 (10) | 2 (4) | 12 (13) | 0.139 |
| 24-hour blood pressure measurement | 9 (6) | 1 (2) | 8 (9) | 0.164 |
| Duplex Carotid Arteries | 4 (3) | 1 (2) | 3 (3) | >0.999 |

**Supplemental table 1.** Detailed characteristics of syncope patients

Values are presented as mean (*SD* standard deviation), median (interquartile range) or as absolute numbers (%). *ACEi* angiotensin-converting enzyme inhibitor, *ARB* angiotensin-receptor blocker, *CT* computed tomography, *LVEF* left ventricular ejection fraction, *LA* left atrium, *LV* left ventricular, *IVST* interventricular septum thickness, *PWT* posterior wall thickness, *MRI* magnetic resonance imaging

**Note:** statistically significant parameters are shown as a bold value.
